# Management of Neuroinflammatory Responses to AAV-Mediated Gene Therapies for Neurodegenerative Diseases

**DOI:** 10.3390/brainsci10020119

**Published:** 2020-02-22

**Authors:** Barbara A. Perez, Alison Shutterly, Ying Kai Chan, Barry J. Byrne, Manuela Corti

**Affiliations:** 1 Department of Pediatrics and Powell Gene Therapy Center, University of Florida, Gainesville, FL 32610, USA; barbara.perez@ufl.edu (B.A.P.); barry.byrne@ufl.edu (B.J.B.); 2 Molecular Genetics and Microbiology, University of Florida, Gainesville, FL 32610, USA; 3 AavantiBio, Inc., Gainesville, FL 32601, USA; alison.shutterly@aavantibio.com; 4 Department of Genetics, Harvard Medical School, Boston, MA 02115, USA; YingKai_Chan@hms.harvard.edu; 5 Wyss Institute for Biologically Inspired Engineering, Harvard University, Boston, MA 02115, USA

**Keywords:** AAV, gene therapy, neurodegeneration, neuroinflammation, immunosuppression

## Abstract

Recently, adeno-associated virus (AAV)-mediated gene therapies have attracted clinical interest for treating neurodegenerative diseases including spinal muscular atrophy (SMA), Canavan disease (CD), Parkinson’s disease (PD), and Friedreich’s ataxia (FA). The influx of clinical findings led to the first approved gene therapy for neurodegenerative disorders in 2019 and highlighted new safety concerns for patients. Large doses of systemically administered AAV stimulate host immune responses, resulting in anti-capsid and anti-transgene immunity with implications for transgene expression, treatment longevity, and patient safety. Delivering lower doses directly to the central nervous system (CNS) is a promising alternative, resulting in higher transgene expression with decreased immune responses. However, neuroinflammatory responses after CNS-targeted delivery of AAV are a critical concern. Reported signs of AAV-associated neuroinflammation in preclinical studies include dorsal root ganglion (DRG) and spinal cord pathology with mononuclear cell infiltration. In this review, we discuss ways to manage neuroinflammation, including choice of AAV capsid serotypes, CNS-targeting routes of delivery, genetic modifications to the vector and/or transgene, and adding immunosuppressive strategies to clinical protocols. As additional gene therapies for neurodegenerative diseases enter clinics, tracking biomarkers of neuroinflammation will be important for understanding the impact immune reactions can have on treatment safety and efficacy.

## 1. Introduction

In recent years, adeno-associated virus (AAV)-mediated gene therapies have gained clinical interest for treating a wide variety of neurodegenerative and neuromuscular diseases including spinal muscular atrophy (SMA) (NCT02122952; NCT03306277, NCT03381729), Canavan disease (CD) [1], Alzheimer’s disease (AD) (NCT03634007), and Friedreich’s ataxia (FA) [2,3,4]. The increased interest in AAV-mediated gene therapies has led to multiple successful clinical trials, with the first approved gene therapy product for a neurodegenerative disease, SMA, in 2019 [5].

The influx of new findings from multiple gene therapies undergoing preclinical and clinical testing has highlighted new hurdles for treatment efficacy and safety concerns for patients. An incomplete understanding of disease pathophysiology, limited access to target tissues within the central nervous system (CNS), and complex disease presentations makes therapeutics development and outcome measurements difficult for disorders of the CNS. Additionally, early works in the field demonstrated that AAV-mediated gene therapies elicit a strong host immune response [6,7,8], resulting in safety concerns for the patient, decreased transgene expression, and decreased longevity of transgene expression [5,6,7,9]. Advances in vector design [10,11,12,13], manufacturing technology [14], and the addition of immunomodulatory treatments to clinical protocols [6,15,16] have focused on controlling immunogenicity of the virus and host immune response to therapy [17,18,19]. 

Since the CNS compartment was believed to be immune-privileged, the development of immunomodulatory strategies has focused on reducing the immune response to systemic viral dosing. However, preclinical studies in large animal models detect AAV-related markers of neuroinflammation and CNS pathology even after CNS-specific delivery methods [20,21], suggesting that the issue of host immune responses to the vector is not exclusive to systemic dosing strategies. While most of these reports show no phenotypic consequence of the pathology at the time points tested (typically 1–3 months post-dose), it is important to understand triggers (e.g., route of delivery, manufacturing impurities in vector material, transgene overexpression), mechanisms of the inflammation (e.g., infiltration of peripheral immune cells, immune reaction within the CNS, transgene-mediated neurotoxicity), and the long-term effects of neuroinflammation. 

In this review, we will discuss neuroinflammation related to AAV-based gene therapies, including CNS-targeting delivery strategies, management of inflammatory responses, and strategies to increase safety and efficacy.

## 2. Neuroinflammation in Adeno-Associated Virus (AAV)-Mediated Gene Therapies 

Although the overall immunogenicity of AAV-based gene therapies is well characterized [6,7,9], immunogenicity from CNS-directed AAV delivery has not been widely investigated. This is partially due to the broadly held belief that the brain and spinal cord reside in an immune privileged compartment, protected by the blood–brain barrier (BBB) [22]. However, recent gene therapy studies for CNS disorders reveal a significant involvement of the immune system in the brain [23,24] and unexpected immune reactions have been reported in several studies of AAV delivery directly into the CNS compartment [25,26]. 

Neuroinflammation is already a feature of many neurodegenerative diseases such as AD, Parkinson’s disease (PD), and multiple sclerosis (MS) [27]. Neuroinflammation involves CNS resident cells (e.g., microglia, astrocytes, oligodendrocytes), peripheral immune cell infiltrates (T cells), breakdown of the blood–brain barrier, pro-inflammatory cytokines and other mechanisms (for detailed reviews on cell types and mechanisms associated with neuroinflammation, see Ransohoff et al. 2010, 2012, 2015, 2016) [24,27,28,29]. Inflammation can be initiated within the CNS compartment, likely microglial mediated, or from outside of the CNS and mediated by infiltrating myeloid cells. T cells can become activated in the periphery and traffic into the CNS in response to peripheral antigens. B cell-mediated humoral responses can be initiated from the periphery or from within the CNS and in neurodegenerative disease, relevant antibodies are often present in both serum and cerebral spinal fluid (CSF) [24,28,29]. The extent of involvement of each component differs between disease, and the mechanisms involved in neuroinflammation in response to AAV exposure likely differ as well. The effect of exacerbating the immune system by exposure to AAV is unknown but an important consideration especially in this class of diseases.

Because AAV-related neuroinflammation is only just emerging as an important subject in gene therapy, there has not been an emphasis on understanding the cell types and mechanisms involved or on collecting extensive biomarker data as part of clinical trial design. However, with increasing reports of neuroinflammation more recently [30,31], additional biomarkers will need to be incorporated into future trial designs as a way to track and better understand these issues. For example, blood, CSF and neuroimaging are the most commonly used sources of biomarkers in neurodegeneration and neuroinflammation due to their non-invasive nature and versatility [32]. Current AAV-based gene therapy trials typically evaluate neuroinflammation by antibodies against the vector capsid and/or transgene in CSF and blood, T cell response against capsid and/or transgene by enzyme-linked immune absorbent spot (ELISPOT) assays, and the presence of pleocytosis in the CSF after dosing [1,5,9,31]. As of now, neuroimaging is typically included as a marker of disease status to evaluate treatment efficacy but is not leveraged to measure neuroinflammation. For example, magnetic resonance imaging (MRI) could be utilized to evaluate inflammation-related neuropathology such as white matter changes, ventricular enlargement [33], or cell death, and BBB breakdown by measuring leakage of gadolinium to the periphery [34]. Additionally, functional imaging can be used to monitor the activation of resident immune cells and begin to understand the mechanisms behind gene therapy-related neuroinflammation [35,36]. Solutes in the CSF including cytokines, glial fibrillary acidic protein (GFAP), and neurofilament proteins could also be employed for tracking neuroinflammation [37]. Since this is an emerging topic, comprehensive biomarker analysis beginning immediately after AAV dosing would provide important insight into the most predictive biomarkers and the mechanism of AAV-related neuroinflammation. Using this data to understand the specific mechanisms involved will help the field to develop methods for preventing or decreasing vector immunogenicity within the CNS.

### 2.1. Systemic Delivery

Systemic delivery of AAV is the most common route of administration for gene therapy and an effective delivery method for multi-systemic diseases with target tissues within and outside of the CNS (multiple completed and ongoing clinical trials using systemic delivery of AAV include NCT02122952, NCT03368742, NCT03315182, NCT03362502). However, to achieve clinically relevant levels of transgene expression across target tissues, especially within the CNS, this route of administration requires high vector doses. Exposing the host immune system to a greater number of viral particles and possible manufacturing impurities could result in exaggerated immune responses. Additionally, the lessons learned from peripheral immune responses elicited by exposure to high systemic doses of AAV should not be disregarded in the context of neuroinflammation since they could also inform AAV immunogenicity in the CNS. One consideration of the immune reaction to systemic vector delivery is the impact it can have on future exposure to the virus. Systemic delivery (as well as natural environmental exposure) of high vector doses will result in circulating anti-capsid and anti-transgene binding antibodies (bAb) and capsid-neutralizing antibodies (nAb), priming the immune system to detect and neutralize future exposures to the virus, presenting additional immune-related safety concerns [38]. A study of prior-immunization in rats demonstrates that a prior exposure to AAV causes high levels of circulating anti-capsid nAbs that completely block viral transduction from a follow-up CNS-specific AAV administration [39]. Additionally, patients with null mutations where the immune system is naïve to endogenous protein, also known as cross-reactive immunological material-negative (CRIM-) patients, perceive the AAV-derived transgene as immunologically foreign and develop higher antibody levels and more severe T-cell responses against the transgene product [19,40]. Clinical trials are successfully implementing additional immunosuppression strategies to limit adaptive anti-transgene immune responses in CRIM- patients [16,30,31], thus increasing the safety and efficacy of AAV-based therapies within this patient population.

Despite its limitations, systemic dosing has been safely and effectively implemented in preclinical and clinical studies for several neurodegenerative diseases requiring viral transduction in the CNS. In a preclinical study of gene replacement therapy for mucopolysaccharidosis (MPS) IIIB using a non-human primate (NHP) model, Murray et al. achieved transgene expression within the CNS after systemic injections of 1 × 10^13^ or 2 × 10^13^ vg/kg [41]. The authors detected anti-AAV9 and anti-transgene antibodies in the circulation of treated animals, but antibody levels did not correlate with decreased transgene expression in the CNS [41]. Systemic administration of AAV9 at doses of 2–5 × 10^13^ vg/kg is being employed in ongoing clinic trials of MPS IIIB (NCT03315182) and MPS IIIA (NCT04088734) with the release of safety and efficacy data unavailable at the time that this review was written. Similarly, several preclinical studies and clinical trials [5,42] evaluated safety and efficacy data with no significant findings in support of the U.S. Food and Drug Administration (FDA) approval of Zolgensma, a systemically delivered gene therapy treatment for children with SMA, which was determined to be safe and effective at a single systemic dose of 1.1 × 10^14^ vg/kg [43].

Ongoing clinical trials for AAV products manage the risk of inflammation by excluding individuals with pre-existing nAbs against the viral capsid, whether it be from a natural environmental exposure or a previous sub-therapeutic dose of gene therapy (e.g., NCT03368742, NCT03315182, NCT03362502, NCT02240407). The currently accepted exclusion criteria leaves out 20–70% of otherwise eligible patients [3,44]. Additionally, multiple subjects who screen negative for pre-existing anti-AAV antibodies still experience complications, suggest that the current criteria is an incomplete measure of immune status [45,46,47]. Major limitations associated with this approach are that standardized laboratory tests have not been established for measuring pre-existing anti-AAV immunity across trials or to better define appropriate markers and thresholds for true immunologically-naïve status. Although not routine for all trials, some are also considering subjects’ CRIM status and pre-existing T cell-mediated adaptive responses in their inclusion criteria and for selection of most appropriate immune interventions [31].

### 2.2. Central Nervous System (CNS)-Directed Delivery

CNS-directed deliveries are a compelling alternative to reduce the overall immune response because they require lower doses of vector to reach clinically relevant transgene expression in CNS tissue. The most heavily researched strategies for CNS-directed AAV delivery are intracerebroventricular (ICV), intra-cisterna magna (ICM), intrathecal (IT), and intraparenchymal injections. ICV, ICM, and IT injections deliver vector into the CSF circulation via lateral ventricle, cisterna magna, or lumbar spinal cord space, respectively. The intraparenchymal route delivers the vector directly into the target brain tissue using stereotactic surgery. These CNS-directed delivery strategies, however, are not enough to completely prevent the effect of circulating anti-AAV antibodies and delivery strategies can physically disrupt the BBB allowing even greater access for circulating antibodies to enter the brain and neutralize the vector [39,48]. 

Numerous preclinical studies of CNS-directed AAV administration have reported circulating nAbs against the vector capsid and elevated markers of cytotoxicity [20,25,49,50]. One study using intra-cisterna magna (ICM) delivery of AAV9 in rhesus macaques detected transgene-binding and AAV9-neutralizing antibodies in the serum and CSF of animals and anti-transgene T-cell responses regardless of the dose administered (1 × 10^12^ and 1 × 10^13^ vector genomes (vg)). [20] Treated animals were asymptomatic but demonstrated bilateral histopathology in the DRGs, axons emanating from dorsal spinal cord white matter, and trigeminal nerve ganglia including mononuclear cell infiltration (mostly CD20+ and CD3+ lymphocytes with few CD68+ macrophages). Mononuclear pleocytosis in the CSF and a transient increase in CSF protein was also reported in AAV-treated but not vehicle-treated animals [20]. Another preclinical study in a NHP model using a combination of intracerebroventricular (ICV) and bilateral intraparenchymal injections of AAV rh8 into the thalamus reported neurotoxicity and associated behavioral changes [25]. Animals treated at three different doses (3.2 × 10^12^, 3.2 × 10^11^, and 1.1 × 10^11^ vg) developed dose-dependent white and gray matter necrosis along the injection track along with dyskinesia and ataxia. Supra-physiological levels of transgene expression were detected in the thalamus and spinal cord of all dosed animals, suggesting neurotoxicity could be associated with overexpression of the transgene. Antibody levels in the CSF were not reported. Finally, studies using dog models of Sanfilippo and Hurler syndrome used intraparenchymal delivery of AAV vector and reported neuroinflammation including lymphocyte, plasma cells, and macrophage infiltration into perivascular and subarachnoid spaces with diffuse hyperplasia and clusters of microglia [49]. Animals who were also treated with immunosuppression agents, cyclosporine (CsA) and mycophenolate mofetil (MMF), had lower incidences of neuroinflammatory findings and increased vector biodistribution [50]. 

In agreement with the studies described, toxicology studies performed by our group on NHP models using combined systemic and IT administration of a human *frataxin (FXN)-*encoding AAV9 vector shows similar histopathological abnormalities. Animals given an IT dose of 1–3 × 10^13^ vg showed spinal cord and DRGs abnormalities including minimal neuronal degeneration/necrosis, minimal to moderate mononuclear cell infiltration, and minimal to mild nerve fiber degeneration of the nerve roots. Two NHP treated with a similar dose of a cynomolgus-specific *FXN*-encoding vector had no findings at a similar dose, suggesting that an anti-transgene immune response could have contributed to the findings of inflammation in this study.

Despite encouraging findings in early preclinical studies that supported commercialization of Zolgensma [51], similar histopathological findings were recently reported in another NHP preclinical study that used IT administration of the SMA gene therapy, AVXS-101 [26]. AVXS-101 is already approved in the US as Zolgensma for systemic use in the treatment of SMA. Zolgensma has not been affected by these findings at the time that this review was written, but the FDA placed a clinical hold on the IT administration trial for subjects with SMA Type 2 (NCT03381729). Low- (6 × 10^13^ vg) and mid-dose (1.2 × 10^14^ vg) cohorts have been completed with no reported clinical findings but the high-dose (2.4 × 10^14^ vg) cohort will not be recruited until further investigation to understand the cases of mononuclear cell infiltration and neuronal degeneration in DRGs of IT-treated NHP [26]. Furthermore, a clinical trial of IT administration of AAV9 in subjects with giant axonal neuropathy (GAN) (NCT03770572) presented findings of elevated markers of neuroinflammation including elevated anti-capsid antibodies and T-cell response and pleocytosis in the CSF [31].

On the other hand, many studies have shown no evidence of neuroinflammation [1,52,53], highlighting the need to compare experimental designs including the appropriateness of a large animal model in preclinical trials and biomarker selection across preclinical and clinical trials. These observations represent a gap in knowledge regarding the mechanisms of AAV immunogenicity in the CNS and the currently employed strategies to modulate it.

## 3. Managing AAV-Mediated Neuroinflammation

Approaches to decrease both innate and adaptive immune responses against the AAV capsid and/or transgene have been under investigation for many years. Most of the research has focused on improving efficacy of the treatment, enabling pre-exposed individuals to receive AAV-based treatment, and allowing for repeated administrations of the vector throughout an individual’s lifetime. To date, the effects on neuroinflammation specifically have not been tested, but the immune modulating approaches reported warrant further characterization for their specific effect on CNS immune reactions. 

### 3.1. Choice of AAV Capsid Serotype and Promoter 

Characterization of first-generation capsids AAV 1, 2 and 5 display low levels of expression and variable cell-type specificity in the CNS. For example, AAV1 and 5 can transduce neurons and glial cells while AAV2 transduces neurons only but have limited spread within the CNS [54]. In 2002, AAV 7, 8, 9 and rh10 were discovered in primates [55]. When the different serotypes are cross- packaged with identical AAV2 genomes, they show variations in cell-type transduction efficacy and affinity to CNS substructures after systemic delivery, making each uniquely suitable for particular disease indications [56,57,58,59,60]. However, most naturally isolated and commonly used AAV vectors only minimally cross the BBB after a systemic injection. The limited amount of vector that does reach the CNS shows a strong neuronal tropism and negligible transduction of glial cells. 

Of the common capsids currently available, AAV9 has emerged as the most widely used for CNS gene therapy applications due to its enhanced spread across CNS structures and its ability to penetrate the BBB even after peripheral administration in neonatal animals [61,62]. While both AAV9 and rh10 show high transgene expression throughout the brain including spinal cord regions, AAV9 shows the greatest rostral-caudal distribution and spreads to the contralateral (un-injected) hemisphere by undergoing axonal transport [57]. Studies in animal models of SMA [42], amyotrophic lateral sclerosis (ALS) [63], MPS IIIB [64], and others show that a systemic injection of AAV9 in neonatal mice results in transgene expression across key CNS substructures and neuronal subtypes such as motoneurons along with improvements in disease-related phenotypes. However, systemic injections in adult mice still result in limited expression and a shift in target cell subtypes, with preferential expression in astrocytes over neurons [61,65,66]. Possible explanations for different viral tropism and spread during stages of development include structural development of the CNS restricting distribution of the virus, changes in expression of capsid internalization receptors, or neurogenesis resulting in enriched vector distribution to the newly emergent cell types. 

Additional discrepancies in cell type tropism and biodistribution between AAV serotypes in the CNS are noted across species and animal models of disease. For example, the PHP.B serotype is an engineered capsid derived from AAV9 that showed remarkable CNS tropism in initial experiments in C57BL/6J mice [67]. Follow up experiments determined that the exceptional neurotropic properties are exclusive to the C57BL/6J mouse strain and not recapitulated in other mouse strains or in NHP [68]. Similarly, differences in blood-brain barrier permeability between healthy animals and models of neurodegenerative diseases could result in different AAV biodistribution in the CNS, possibly contributing to discrepancies in translating findings from mouse models of disease into toxicology studies using larger, healthy animal [69]. 

In addition to choice of capsid for safest and most disease-relevant spatial and temporal requirement for transgene expression, alterations to the transgene-coding region and regulatory elements are also common. Most clinical studies currently use high-expressing ubiquitous promoters such as variants of the human cytomegalovirus (CMV) or chicken beta-actin (CBA) promoters with CMV enhancer (CAG) [70]. Variations of this promoter have been characterized extensively and show robust expression throughout neuronal cell types in the CNS [71]. The incorporation of transgene-specific regulatory elements such as the endogenous transgene promoter and/or the 3’ untranslated region (UTR) [72,73] is a possible strategy for reducing neurotoxicity from overexpression or expression in non-target cell populations [74]. The transgene-coding sequence can also be optimized in a variety of ways including codon optimization for enhanced expression in a particular species. When validating constructs for human transgene expression in preclinical models such as rodents or NHP, additional variables should be considered such as expression level differences in the test model and possible cross-species anti-transgene reactivity. 

Thoughtful selection of capsid and transgene expression elements in early experimental design stages might support smoother clinical translation by preventing the need to use more invasive delivery methods and immunosuppression strategies. Ongoing preclinical studies in large animals including comparison experiments using co-delivery of vectors with identifying barcodes and future clinical trials will continue to inform on these important capsid tropism differences.

### 3.2. Route of Administration 

**Figure 1:**
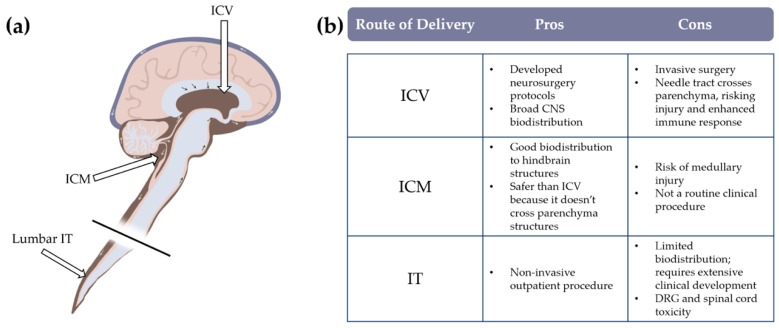
Comparison of central nervous system (CNS)-directed dosing strategies. (**a**) Schematic diagram of relative location of cerebrospinal fluid (CSF)-targeting injections. (**b**) Table of pros and cons between common routes of CSF delivery.

While systemic administration safely and effectively delivers a large amount of virus across multiple tissues, it does not effectively penetrate the BBB in adults. An additional disadvantage of this administration route for CNS disorders is that delivering large amounts of virus into the circulation resulting in unnecessary exposure to the virus, risking greater host immune response. For example, experiments comparing equivalent doses of AAV9 (total vg exposure) administered either systemically or directly into the CSF resulted in dramatically enhanced transduction efficacy in CNS and sensory neurons in direct CSF delivery compared to systemically administered vector [58]. In this study, a 50-fold decrease in CSF-administered dose was sufficient to achieved similar neuronal transduction in DRGs compared to systemic administration [58]. Intracerebroventricular (ICV), intra cisterna magna (ICM), and intrathecal (IT) injections are three widely recognized strategies to delivery drugs into the CSF circulation (Figure 1). Direct intraparenchymal injections can also be used for more selective targeting of specific brain regions and to limit spread within the CNS. 

An ICV injection consists of delivering the drug directly into the CSF through the lateral ventricles providing the broadest CNS distribution. Although this technique is relatively safe and effective, and is routinely undertaken by neurosurgeons [75], it is not without risks and complications including infections, intracerebral hemorrhage, subcutaneous CSF leaks and increased intracranial pressure [75,76,77,78]. However, these rare complications are most often associated with chronic delivery of biologics, and single-delivery AAV treatments will likely be safer.

An ICM injection delivers the virus to CSF via the cisterna magna, located below the fourth ventricle and between the cerebellum and medulla, resulting in more directed viral exposure to the cerebellum, brainstem, and spinal cord compared to ICV. After a single ICM injection in a feline model of MPS I, Hinderer et al. reported significant transgene expression at comparable levels across cortex, hippocampus, medulla, cerebellum, and spinal cord regions [79]. However, this approach is rarely utilized in clinical practice and would need significant procedural development to safely enter clinical trials due to the route’s increased risk for medullary injury and related complications [80]. 

Lumbar IT injections are routine clinical procedures with few complications that are used to safely access CSF for biomarker measures and drug delivery. A mouse experiment comparing equivalent amounts of AAV9 dosed either systemically or by an IT injection shows that the IT route results in robust transduction across the CNS including spinal neurons, sensory neurons, and DRGs at every level of the spinal cord [58]. IT delivery of AAV is being tested across several clinical trials for SMA (NCT03381729), GAN (NCT03770572), MPS II (NCT0356604), and others. CSF is generated by the choroid plexus in the lateral ventricles, and flows downward through the third and fourth ventricles, down to the lumbar cistern in the spinal cord [81]. Thus, delivering virus through a lumbar IT injection provides the least amount of spread, requiring thoughtful consideration of fluid dynamics within the CSF to achieve improved CNS biodistribution. For example, Meyer et al. report that maintaining NHPs in a Trendelenburg position for 5–10 min after the IT dosing improves viral transduction in the brain and brainstem [51]. This approach has been incorporated into the IT delivery method in a clinical trial of GAN subjects (NCT02362438).

Finally, although intraparenchymal injections are more invasive [9,82] and result in a limited coverage of the CNS, this approach is likely immunologically safest, requiring the least amount of virus to reach clinically relevant transgene expression if target tissues are few and easy to isolate. This approach is best suited for indications with a well-recognized site of CNS pathology such as Huntington’s disease (HD), Parkinson’s disease (PD), and Canavan disease (CD). For example, in a mouse model of HD, bilateral injections of AAV5 carrying a microRNA targeting *huntingtin (HTT)* into the striatum resulted in reduction of toxic HTT protein and improved motor function in treated animals [83]. In the clinic, intraparenchymal dosing of AAV for the treatment of neurodegenerative diseases has shown acceptable safety profiles across several trials. A Phase I trial using bilateral injections of AAV2-AADC into the putamen of PD patients was well tolerated and resulted in improved AADC expression and motor function one-year post-dosing [84]. Another Phase I trial using six cranial burr holes to deliver a gene therapy treatment to subjects with Canavan disease also showed minimal systemic immune reactions with no overt neuroinflammation [9]. 

### 3.3. Genetic Manipulations to Decrease TLR9-Mediated Immune Responses

Toll-like receptors (TLRs) are pattern recognition receptors found on the endosomes of immune cells that play a role in the detection of pathogens and the initiation of innate immune and inflammatory responses, including type 1 interferon and pro-inflammatory cytokines [85]. TLR9 has been implicated in immune recognition of AAVs by binding to unmethylated CpG motifs in the AAV genome and activating signaling adaptor protein, myeloid differentiation primary response gene 88 (MyD88) [86]. Activation of the TLR9-MyD88 pathway subsequently promotes the development of CD8+ cytotoxic T cell responses against AAV capsid and transgene, which can result in loss of transgene expression.

Significant research has focused on characterizing the TLR9-mediated immune response to AAV-vector DNA and finding ways to ameliorate it. First, experiments comparing AAV-treated TLR9 knockout (TLR9-/-) and wild type mice support the direct involvement of the TLR9 pathway in immune activation and transgene loss [10]. When TLR9-/- and wild-type mice received intramuscular (IM) injections of an immunogenic AAVrh32.33 vector, the authors found that wild-type animals exhibited extensive immune cell infiltration into muscle tissue and that transgene expression was eventually lost. In contrast, TLR9-/- mice showed diminished immune cell infiltration and retained persistent transgene expression. Similar outcomes were seen in a follow up experiment performed in wild type mice dosed with the same vector or a CpG depleted version. However, it is unclear how translatable this strategy is to the clinic since CpG motifs are present in both the transgene encoding region and necessary regulatory elements such as the viral ITRs, promoter, and introns. Second, Martino et al. reported another contributing factor to the extent of the TLR9-dependent response to the AAV genome—that single stranded DNA viral genomes are less immunoreactive than double stranded (self-complementary) viral genomes when tested with intravenous injections in mice [87]. Finally, an approach presented at the 2019 American Society for Genetic and Cell Therapies (ASGCT) Annual meeting directly incorporated TLR9 inhibitory oligonucleotide sequences into an untranslated region of the vector genome to “cloak” vector DNA from stimulating TLR9 [12]. The group reported that following IM injection of AAVrh32.33 vectors, mice receiving the modified vector showed lower levels of CD8+ T cell infiltration into muscle tissue compared to the unmodified vector [12].

Since the robust TLR9-mediated responses to AAV were discovered from systemic or IM administration of the virus, most of the work done to understand and prevent immune recognition have also been performed outside of the CNS. As such, the role of TLR9-signaling in AAV-related neuroinflammation for CNS applications is still not well characterized. Analogous to direct CNS administration, the strategy of direct incorporation of TLR9-inhibitory oligonucleotides into the AAV vector genome was also tested in large animals via intraocular administration. The authors observed that subretinal injections of pigs with the unmodified AAV8 vector stimulated photoreceptor pathology, microglia infiltration into the photoreceptor layer and CD8+ T cell infiltration into the retina, while the modified vector evaded such pathology and immune cell infiltration [13]. These findings suggest that TLR9 may play a key role in AAV-mediated neuroinflammation in the CNS. TLRs are expressed in neurons, microglia and astrocytes with TLR9 expression predominantly in microglia [88,89,90,91]. TLR9 has been implicated in mediating the innate immune response to herpes simplex virus infection in the brain [92] as well as in pathobiology of several neurodegenerative diseases [91,93]. 

In an experiment to understand the neuroinflammatory effect of TLR9 activation, mice received an ICV dose of a CpG-containing oligodeoxynucleotide (CpG-ODN), a TLR9 agonist. A single low dose of TLR9 agonist induced signs of neuroinflammation including severe meningitis, increase in proinflammatory cytokines and chemokines, a breakdown of the BBB, and infiltration of immune cells from the periphery [94]. Although the TLR9 agonist used in these experiments exposed animals to much larger amounts of total nucleic acid than a typical AAV dose, this work highlights risks associated with TLR9 activation in the CNS and encourages therapeutic development to consider the immune-evasive strategies presented here as well as the identification of other strategies to modulate the TLR9 response to AAV within the CNS.

### 3.4. Immunomodulation Strategies and Their Effect on CNS 

Even very low titers of anti-capsid antibodies can completely block the therapeutic effect of AAV administration in the CNS. AAV treatment has been associated with anti-capsid and anti-transgene circulating antibodies in blood and the CSF as well as infiltrated mononuclear cells in CSF, and neural tissue, thus highlighting a need for managing antibody-based and cell-based responses to AAV treatment. Most clinical trials now incorporate peri-procedural corticosteroids, and others include additional immunosuppressive agents such as B cell depleting rituximab and mTor regulating rapamycin [1,15]. A single subject case report by our group showed that immune modulation with rituximab and rapamycin prior to AAV administration blocked antibody-based immune responses to both capsid and transgene [15].

Plasmapheresis has been proposed as a strategy for complete removal of circulating antibodies because it would increase safety of dosing and possibly allow for the participation of pre-immune individuals in AAV-based gene therapy treatments. In a study of 10 subjects undergoing plasmapheresis, anti-capsid nAbs against AAV serotypes 1, 2, 6, and 8 were measured before and after each treatment [95]. Between 1–20-fold decreases in nAb titer were noted after each round of plasmapheresis for all serotypes analyzed. However, a “rebound” effect was observed where nAbs return to previous levels after the treatment, and even after five treatments, nAb titers fell below the cutoff criteria in only two of the 10 subjects. Importantly, those two subjects already had the lowest titers at study baseline, suggesting this approach is feasible for managing only low or moderate levels of preexisting immunity to AAV [95]. Another study used naturally exposed AAV-preimmune NHPs to evaluate the effect of two rounds of plasmapheresis on nAb titer. In contrast to the clinical findings summarized above, this preclinical study reports that the nAb titers were reduced to levels similar to the naïve animals after only two treatments in all seven NHP models treated [96]. There are still few studies on combining plasmapheresis with AAV-based gene therapy, and additional work is required to understand the best application of the technique.

In addition to managing systemically circulating antibodies, difficult to eliminate long-lived plasma cells may be reactivated by AAV treatment, secreting additional antibodies [97,98]. Plasma cells are highly resistant to most currently available immunosuppressive strategies, with stem cell transplants with anti-thymocyte globulin treatment being the most effective but presenting significant safety risk to patients [96,99,100]. Pre-treatment with plasma cell-targeting agents such as bortezomib might be useful in depleting the plasma cell population and decreasing reactivation upon subsequent AAV exposure [3,101]. Recent work suggests that microglia may also form an immunological memory similar to plasma cells [83], suggesting that long-lived immunological memory may be a concern within the CNS compartment as well. Although the effectiveness of bortezomib on neuroinflammation has not been evaluated, other agents are beginning to be tested for CNS application. For example, mTor modulator rapamycin has been shown to have specific effects on neuroinflammation. A study using in vitro and mouse models of spinal cord injury shows that treatment with rapamycin results in neuronal survival, reduced inflammation, and astrocyte proliferation after spinal cord injury [102]. 

While limited in scope, this data supports a role for broad immunosuppressive strategies in attenuating neuroinflammation in CNS-targeting gene therapies. Additional work is warranted to identify which agents have the best safety profiles and are effective within the CNS compartment.

## 4. Conclusion

**Figure 2:**
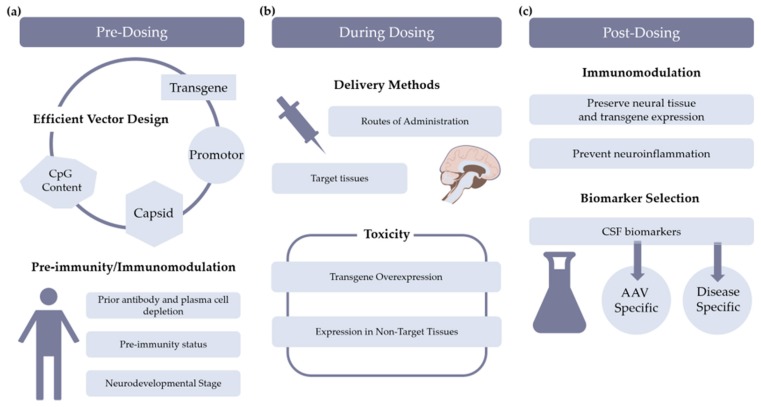
Schematic overview of possible strategies to mitigate immune responses at various stages during gene therapy. (**a**) Pre-dosing considerations include vector design and treatment strategies to prepare the immune system for adeno-associated virus (AAV) exposure. (**b**) During dosing of the vector, the route of administration is selected in consideration of disease-relevant target tissues as to minimize toxicities from transgene overexpression or transgene expression in non-target tissues. (**c**) Post-dosing considerations include selection of immune management strategies that protect trangene expression and neural tissue and decrease neuroinflammation. .

The neuroinflammatory reaction to AAV-based gene therapies for CNS diseases is still not well characterized. However, preclinical and clinical findings in recent years indicate significant vector-related immune reactions and neuroinflammation in subjects. Several strategies to modulate immune-related vector toxicities were discussed and are summarized in Figure 2. Based on the specific disease indication, transgene, and target tissue, few or all of these strategies should be considered to enhance patient safety. The inclusion of biomarkers to evaluate neuroinflammation at key time points will be critical to meet this aim.
